# Non-target site-based resistance to tribenuron-methyl and essential involved genes in *Myosoton aquaticum* (L.)

**DOI:** 10.1186/s12870-018-1451-x

**Published:** 2018-10-11

**Authors:** Weitang Liu, Shuang Bai, Ning Zhao, Sisi Jia, Wei Li, Lele Zhang, Jinxin Wang

**Affiliations:** 1 Key Laboratory of Pesticide Toxicology and Application Technique, College of Plant Protection, Shandong Agricultural University, Taian, 271018 Shandong China; 2 Taian Customs, Taian, 271000 Shandong China

**Keywords:** Acetolactate synthase, Non-target site-based resistance, Metabolic resistance, RNA-Seq, Tribenuron-methyl, *Myosoton aquaticum* (L.)

## Abstract

**Background:**

Water chickweed (*Myosoton aquaticum* (L.)) is a dicot broadleaf weed that is widespread in winter fields in China, and has evolved serious resistance to acetolactate synthase (ALS) inhibiting herbicides.

**Results:**

We identified a *M. aquaticum* population exhibiting moderate (6.15-fold) resistance to tribenuron-methyl (TM). Target-site ALS gene sequencing revealed no known resistance mutations in these plants, and the in vitro ALS activity assays showed no differences in enzyme sensitivity between susceptible and resistant populations; however, resistance was reversed by pretreatment with the cytochrome P450 (CYP) monooxygenase inhibitor malathion. An RNA sequencing transcriptome analysis was performed to identify candidate genes involved in metabolic resistance, and the unigenes obtained by de novo transcriptome assembly were annotated across seven databases. In total, 34 differentially expressed genes selected by digital gene expression analysis were validated by quantitative real-time (qRT)-PCR. Ten consistently overexpressed contigs, including four for CYP, four for ATP-binding cassette (ABC) transporter, and two for peroxidase were further validated by qRT-PCR using additional plants from resistant and susceptible populations. Three CYP genes (with homology to *CYP734A1*, *CYP76C1*, and *CYP86B1*) and one ABC transporter gene (with homology to *ABCC10*) were highly expressed in all resistant plants.

**Conclusion:**

The mechanism of TM resistance in *M. aquaticum* is controlled by NTSR rather than TSR. Four genes, *CYP734A1*, *CYP76C1*, *CYP86B1*, and *ABCC10* could play essential role in metabolic resistance to TM and justify further functional studies. To our knowledge, this is the first large-scale transcriptome analysis of genes associated with NTSR in *M. aquaticum* using the Illumina platform. Our data provide resource for *M. aquaticum* biology, and will facilitate the study of herbicide resistance mechanism at the molecular level in this species as well as in other weeds.

**Electronic supplementary material:**

The online version of this article (10.1186/s12870-018-1451-x) contains supplementary material, which is available to authorized users.

## Background

Herbicide resistance in weeds-which has evolved as an adaptation to herbicide stress-is a global problem threatening crop production. Resistance to herbicides occur via target-site- and non-target site-based resistance mechanisms (TSR and NTSR, respectively). TSR arises from amplification of a target enzyme or structural changes in the herbicide binding site, is widespread in weeds. NTSR minimizes the amount of herbicide that reaches the target site [1–7]. Enhanced herbicide metabolism (i.e., metabolic resistance) by enzymes such as cytochrome P450 (CYP) monooxygenase, glutathione-S-transferase (GST), glycosyltransferase (GT), ATP-binding cassette (ABC) transporter, peroxidase (POD), esterase, and hydrolase contributes to NTSR [1, 3, 8]. Plants may develop metabolic resistance to existing or novel herbicides, posing a major challenge for weed management [3, 8].

Acetolactate synthase (ALS)-inhibiting herbicides are applied to a variety of crops worldwide. However, their overuse has resulted in the emergence of resistant weeds. At least 160 weed species are currently known to exhibit resistance to ALS inhibitors, which is more than to any other type of herbicide [9]. TSR is the most common mechanism of resistance to ALS inhibitors [7, 10]; to date, at least 28 amino acid substitutions at eight positions (Ala122, Pro197, Ala205, Asp376, Arg377, Trp574, Ser653, and Gly654) of the *ALS* gene have been identified in various weed species [10, 11]. However, in grass weeds, NTSR is considered as the major mechanism of resistance to ALS and acetyl-coenzyme A carboxylase (ACCase) inhibitors [1], although there are few documented cases among dicot weeds [1, 10].

TSR and NTSR can co-evolve under selective pressure from herbicides; thus, the two mechanisms may coexist in a single species, population, or individual [12, 13]. Genes involved in metabolic resistance can vary according to species and history of herbicide application [3, 10]; however, in most cases, the underlying genetic mechanisms have not been elucidated.

RNA-sequencing (RNA-Seq) technology has been used to investigate the genetic basis of abiotic stress responses in plants [14, 15], especially in non-model species for which genomic resources are unavailable [1, 16]. It has also been applied in studies on herbicide resistance in weeds and on genetic differences between resistant and susceptible plants [17]. Genes involved in NTSR to different herbicides have been identified by RNA-Seq in grass weeds such as ryegrass (*Lolium rigidum* Gaudin) [18–20], black-grass (*Alopecurus myosuroides* Huds.) [21], American sloughgrass (*Beckmannia syzigachne* Steud.) [22], shortawn foxtail (*Alopecurus aequalis* Sobol.) [23], *Brachypodium hybridum* [24], perennial ryegrass (*Lolium perenne*) [25], and flixweed (*Descurainia sophia* L.) [26].

Water chickweed (*Myosoton aquaticum* (L.)) is a diploid dicot broadleaf weed that is widespread in winter fields in China and has evolved resistance to many ALS inhibitors [27]. Previous studies have reported *ALS* mutations in *M. aquaticum*, identifying TSR as the major mechanism of herbicide resistance although coexisting NTSR was observed in many populations [28, 29]. Here, we identified a tribenuron-methyl (TM)-resistant population of *M. aquaticum* (HN10) with no known ALS resistance mutations in surviving individuals. We obtained resistant plants from the HN10 population through two rounds of selection (designated MR) and identified the genes involved in TM resistance by RNA-Seq and quantitative real-time (qRT-)PCR analyses.

## Results

### TM dose-response in the absence and presence of malathion

**Fig. 1:**
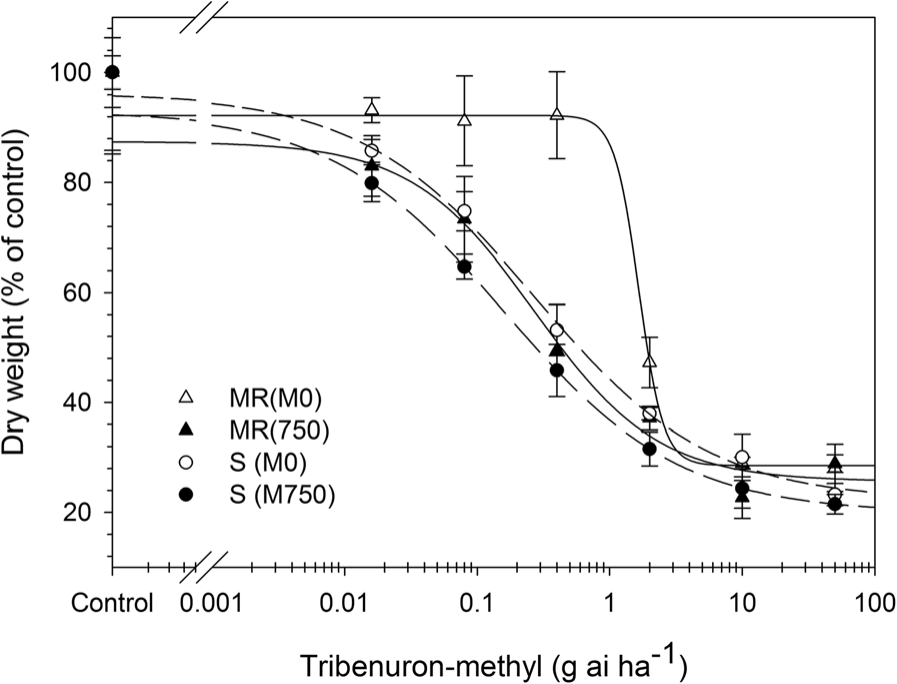
Dose–response curves of susceptible (S) and resistant (MR) *Myosoton aquaticum*. Populations to tribenuron-methyl in the absence (M0) and presence of 750 g ai ha^− 1^ malathion(M750). The solid lines for MR population, and the dash lines for S population. Each data point is the mean ± SE of two experiments

Dose-response studies confirmed that the MR *M. aquaticum* population showed moderate resistance to TM (6.15-fold higher than S plants). When malathion was applied at 750 g ha^− 1^, there was no effect on plant biomass (Table 1 and Fig. 1) in either the S or MR populations; however, the MR population developed sensitivity to TM and the sensitivity of the already susceptible S population was further enhanced (Table 1 and Fig. 1). Malathion is a CYP inhibitor [30, 31] that has been widely used as a marker of CYP involvement in metabolic resistance to ALS inhibitors [10]. In the present study, treatment with malathion combined with TM increased the sensitivity of the MR population, likely through enhanced metabolism mediated by CYP.

**Table 1:** GR_50_ and I_50_ values of the susceptible (S) and resistant (MR) *Myosoton aquaticum* population for tribenuron-methyl

Herbicide	GR_50_ (g ai ha^−1^)^a^			I_50_ (μM)^b^		
	S	MR	RI^c^	S	MR	RI^c^
Tribenuron-methyl	0.27 ± 0.09	1.66 ± 0.72	6.15	0.34 ± 0.05	0.42 ± 0.17	1.24
Tribenuron-methyl+malathion	0.16 ± 0.09	0.30 ± 0.10	1.88	_	_	_

The level of resistance (RI) indicated by the MR/S ratios. Each value represents the mean (±SE) of two experiments, each containing three replicates

^a^GR_50_, herbicide rate causing 50% growth reduction of plants

^b^I_50_, herbicide concentration causing 50% inhibition of the ALS activity

^c^RI (resistance index) = GR_50_ or I_50_ (MR)/GR_50_ or I_50_ (S)

### In vitro ALS activity assay and *ALS* gene sequencing

**Fig. 2:**
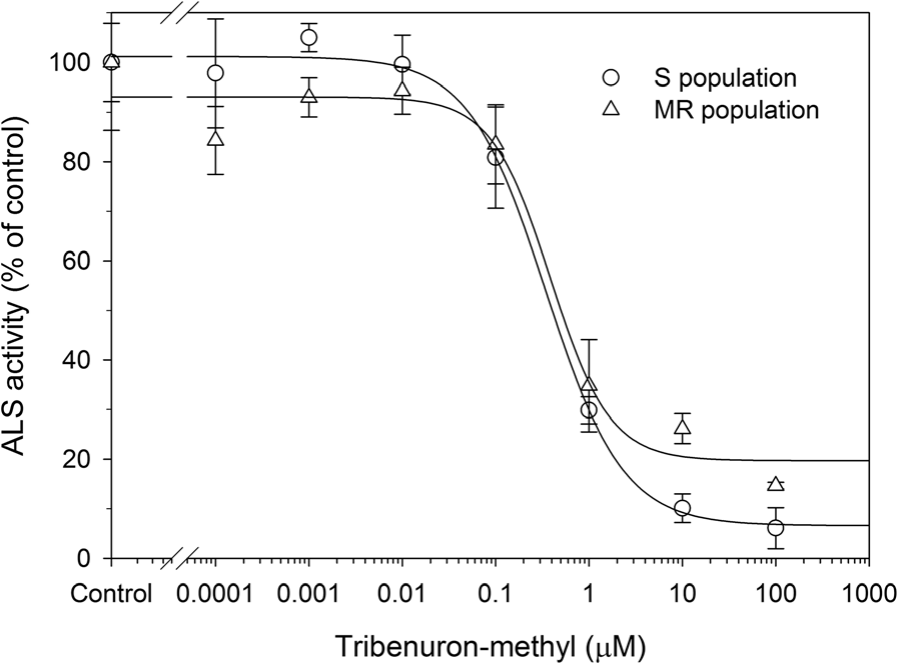
Inhibition of ALS from susceptible (S, ○) and resistant (MR, △) *Myosoton aquaticum* populations by tribenuron-methyl. The ALS activity is expressed as the percentage of activity in the absence of herbicide. The data represent the mean ± SE of two extractions, each containing three replicates

ALS activity in the MR and S populations was compared with an in vitro ALS activity assay. There was no difference in ALS gene expression between the two groups, and gene expression could be induced by TM in both populations (unpublished data). In the absence of TM, total ALS activity was lower in extracts from MR as compared to S plants (11.76 ± 0.53 vs. 21.78 ± 1.72 μmol acetoin formed min^− 1^ mg^− 1^ protein). Adding TM to the reaction almost completely inhibited ALS activity in both S and MR plants at concentrations ≥1.0 μM (Fig. 2). The I_50_ values of S and MR plants were similar (0.34 ± 0.05 and 0.42 ± 0.17 μM, respectively; *P* = 0.8733) (Table 1). *ALS* gene sequencing revealed no known *ALS* resistance mutations in MR plants (GenBank accession nos. of *ALS* gene sequences for S and MR are KF589890.1 and MF288558, respectively). Thus, whole-plant resistance in the MR population is not due to a TSR mechanism.

### RNA-Seq and de novo assembly

To obtain a comprehensive set of transcripts for *M. aquaticum*, a pooled cDNA library of 12 mixed RNA samples from *M. aquaticum* seedlings was analyzed with the Illumina HiSeq 4000 platform. The library generated 603,682,588 raw reads (Table 2). After quality control and data clean-up, we obtained 551,895,330 clean reads ranging from 44,080,630 to 47,652,304 per sample that were then used for de novo assembly (Table 2 and Additional file 1). The reads were assembled into 182,036 transcripts with an average length of 1121 bp; their length distributions are shown in Additional file 2. Up to 94.37% of sequenced clean reads were mapped back onto the transcripts using Bowtie 2; of these, 101,307 unigenes > 200 bp and 44,649 unigenes > 500 bp with a mean length of 872 bp and an N50 length of 1650 bp were obtained using the longest transcript at each locus of each gene (Table 2 and Additional file 2). In addition, the Basic Local Alignment Search Tool (BLAST) matched 35.8% of unigenes to sequences of *Arabidopsis thaliana* (L.) Heynh., with an average accuracy of 71.56%.

**Table 2:** Summary statistics of *Myosoton aquaticum* transcriptome sequencing and assembly

Total raw reads	603,682,588
Total clean reads	551,895,330
Clean base	82.78G
Total assembled transcripts	182,036
No.unigenes> 200 bp	101,307
No.unigenes> 500 bp	44,649
Maximum length	102,348
Minimum length	201
Average unigene length	872
N50 value	1650
N90 value	324
Total nucleotides of unigenes	88,439,116

### Gene annotation and functional classification

Unigene annotation was conducted by BLAST searches against seven public databases (Table 3). In total, 44,117 (43.55%) unigenes were successfully annotated in at least one of the seven databases, and 2276 (2.25%) unigenes were annotated in all seven databases. The greatest sequence similarity was obtained with the Non-redundant database. The annotation results indicate that *M. aquaticum* was most similar to *Beta vulgaris subsp. vulgaris* (30.12% of total unigenes) and *Spinacia oleracea* (15.08%) (Additional files 3, 4).

**Table 3:** Sequence annotation of the *Myosoton aquaticum* transcriptome

Public database	Number of unigenes	Percentage (%)
Annotated in NR	37,948	37.46
Annotated in NT	10,694	10.56
Annotated in KEGG	16,475	16.26
Annotated in Uniprot	33,717	33.28
Annotated in PFAM	24,049	23.74
Annotated in GO	32,395	31.98
Annotated in COG	13,379	13.21
Annotated in all Databases	2276	2.25
Annotated in at least one Database	44,117	43.55
Total unigenes	101,307	100

**Fig. 3:**
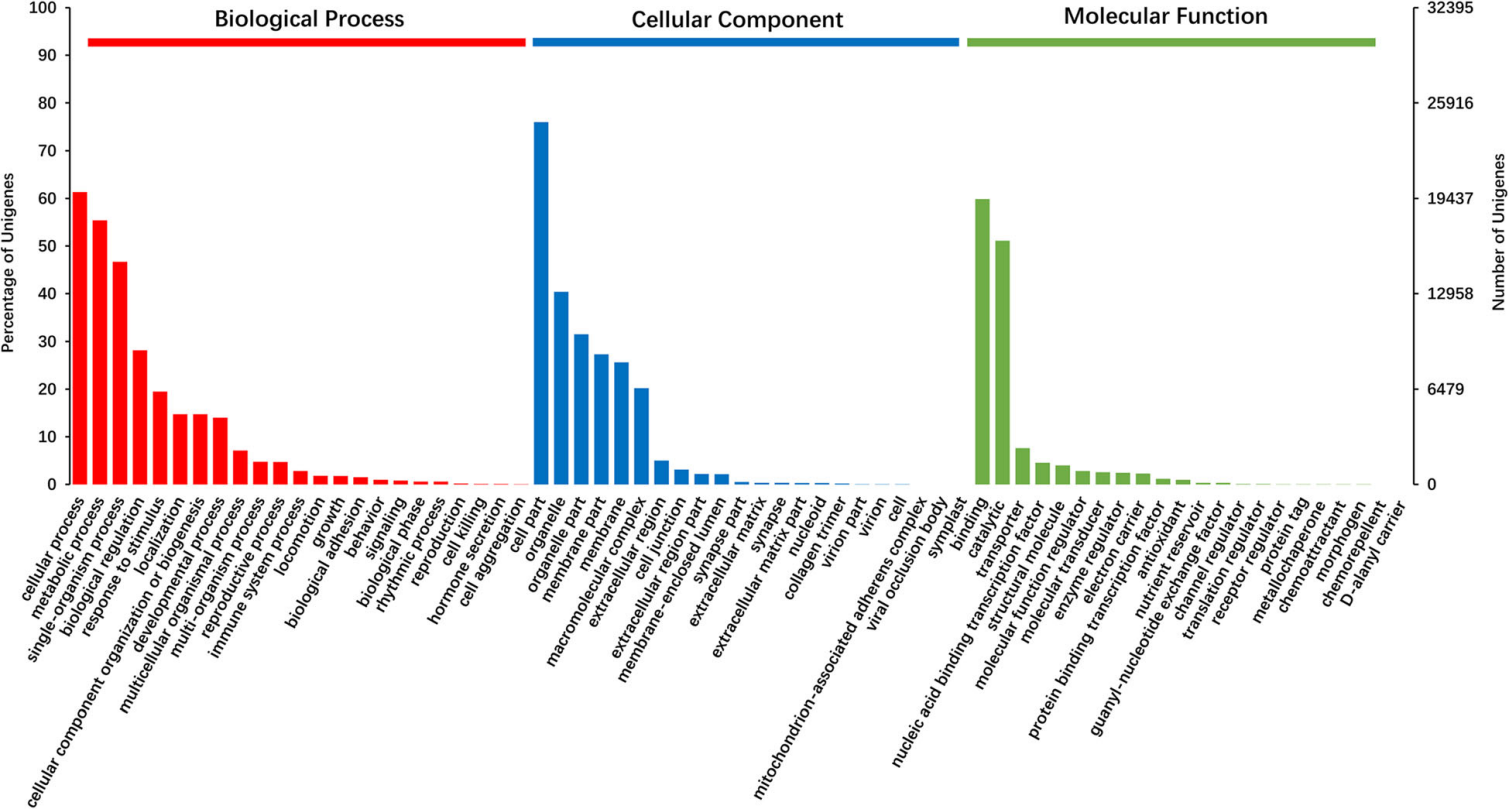
GO function classification of the annotated unigenes in *Myosoton aquaticum*. The unigenes were summarized in biological process, cellular component and molecular function

**Fig. 4:**
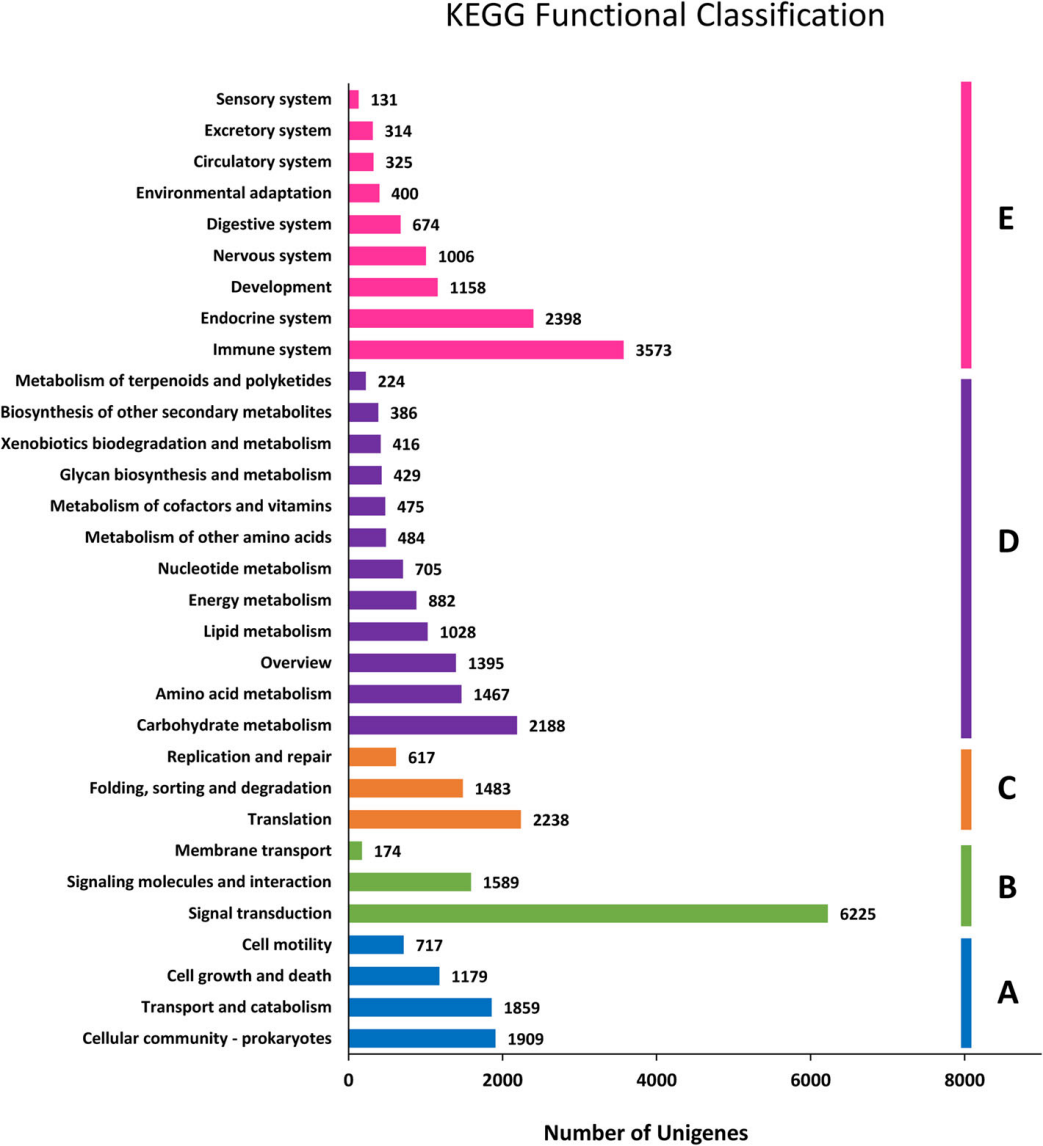
KEGG function classification results of the annotated unigenes in *Myosoton aquaticum*. The y-axis lists the KEGG pathways. The x-axis indicates the number of genes. According to participation in KEGG pathways, unigenes were divided into five groups: A, cellular processes; B, environmental information processing; C, genetic information processing; D, metabolism; E, organism systems

GO and KEGG pathway analyses were performed to predict the functions of annotated unigenes and gene products. In total, 32,395 (31.98%) unigenes were annotated with the GO database, and these were divided into 62 functional subgroups, including 23 for “biological process” (BP), 19 for “cellular component” (CC), and 20 for “molecular function” (MF) (Fig. 3). The largest gene subgroup in BP was cellular process (19,870 unigenes, 61.34%), followed by metabolic process (17,951 unigenes, 55.41%) and single-organism process (15,128 unigenes, 46.70%). In the CC category, cell part (24,626 unigenes, 76.02%), organelle (13,079 unigenes, 40.37%), and organelle part (10,216 unigenes, 31.54%) were the most highly represented. The largest gene subgroup in MF was binding (19,390 unigenes, 59.85%), followed by catalytic (16,550 unigenes, 51.09%) and transporter (2461 unigenes, 7.60%). Additionally, 16,475 unigenes were classified into 31 KEGG pathways comprising five terms, including cellular processes (5664 unigenes, 34.38%), environmental information processing (7998 unigenes, 48.49%), genetic information processing (4338 unigenes, 26.33%), metabolism (10,079 unigenes, 61.18%), and organismal systems (9979 unigenes, 60.57%) (Fig. 4).

### Functional analysis of DEGs

**Fig. 5:**
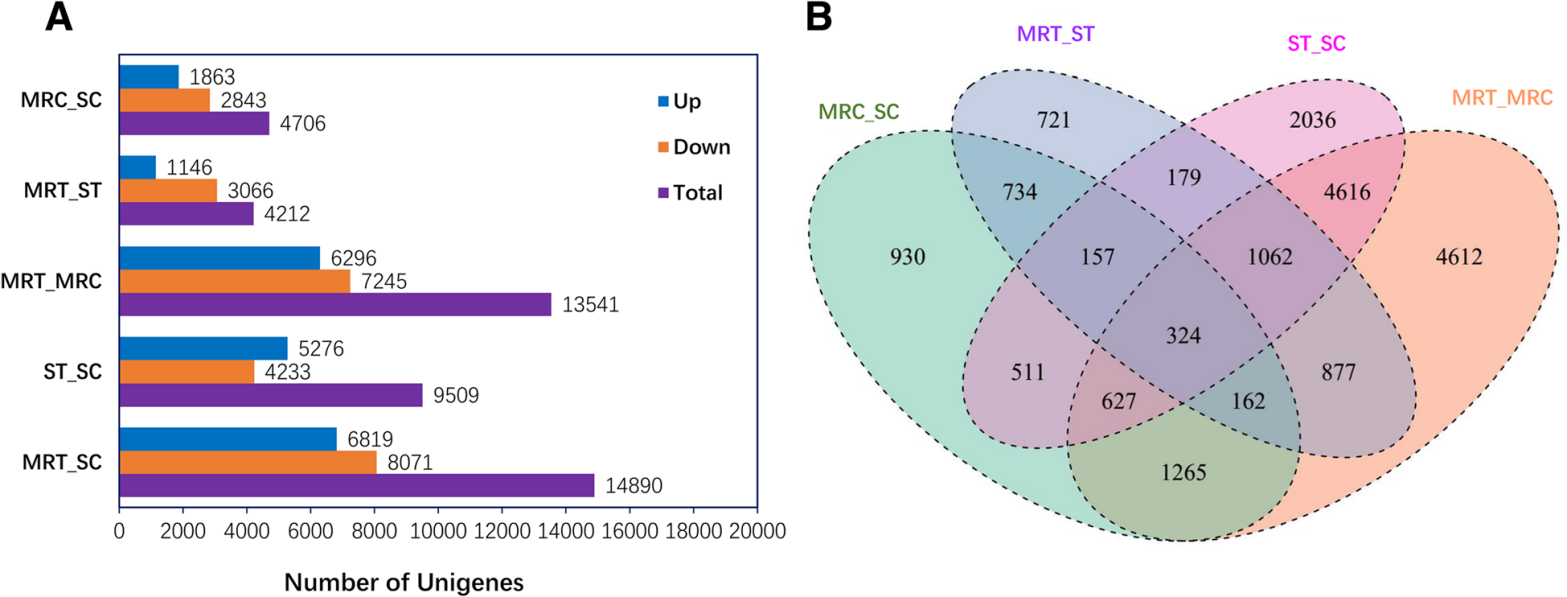
The statistics of the DEGs between the *Myosoton aquaticum* treatment groups. (**a**) The number of DEGs between the different groups^a^. (**b**) Venn diagram showing the number of DEGs between resistant (MR) and susceptible (S) in four treatment comparisons. ^a^The data of ST_SC was cited from another article. Bai S, Liu W, Wang H, Zhao N, Jia S, Zou N, Guo W, Wang J: Enhanced herbicide metabolism and metabolic resistance genes identified in tribenuron-methyl resistant *Myosoton aquaticum* L. J. Agric. Food Chem. 2018;66(37):9850-9857

Transcript levels were calculated according to RPKM. In total, 4706 genes were differentially expressed between untreated MR(MR_C) and S (S_C) samples (|log2 (fold change)| ≥ 1 and q < 0.05), of which 1863 and 2843 were upregulated in MR_C and S_C, respectively. A comparison of TM-treated and untreated samples revealed 1146 and 3066 genes that were upregulated in MR and S, respectively. Meanwhile, 6296 genes were upregulated while 7245 were downregulated in MR_T as compared to in MR_C; and 5276 genes were upregulated while 4233 were downregulated in S_T as compared to S_C (Fig. 5A). Differential expression between MR and S in all four treatments was observed for 324 contigs (Fig. 5B), and 463 overlapping contigs were significantly upregulated in MR_T vs. S_T and in MR_C vs. S_C, including five annotated as CYP, two as ABC transporters, one as GTs, three as POD, and two as esterases (Additional file 5).

To further characterize the function of the identified DEGs, we performed GO and KEGG pathway enrichment analyses. In total, 4212 genes that were differentially expressed between the MR_T and S_T samples were enriched in 53 GO sub-terms (21 BP, 16 CC, and 16 MF). Among these DEGs, the GO sub-terms “cellular process” (1116, 26.50%), “metabolic process” (1037, 24.62%), and “single-organism process” (792, 18.80%) in the BP category; “cell part” (1415, 33.59%), “organelle” (791, 18.78%), and “membrane part” (525, 12.46%) in the CC category; and “binding” (1220, 28.96%) and “catalytic” (1053, 25.00%) in the MF category were enriched in MR relative to S samples. Moreover, the DEGs were enriched in 310 KEGG pathways, with the plant hormone signal transduction pathway enriched in MR and S populations. Among the genes that were differentially expressed between MR_T and S_T samples and were among the top 15 enriched pathways (Table 4), eight and nine upregulated genes were enriched in the “metabolism of xenobiotics by CytP450” and “drug-metabolizing cytochrome CytP450” pathways, respectively. This indicates that CYP genes play an important role in metabolic resistance to TM in *M. aquaticum*.

**Table 4:** The fifteen enriched KEGG pathway terms of the DEGs between tribenuron-methyl treated MR and S *Myosoton aquaticum* populations

KEGG pathway term	Map ID	Gene Count^a^		Genes in back ground^b^	*P*-Value
		Up	Down		
Biosynthesis of secondary metabolites	map01110	94	228	1505	5.94E-09
Plant-pathogen interaction	map04626	64	21	258	1.51E-11
Ribosome biogenesis in eukaryotes	map03008	61	12	217	1.44E-10
Plant hormone signal transduction	map04075	36	48	233	7.67E-14
Phenylpropanoid biosynthesis	map00940	26	40	163	7.23E-14
Phenylalanine metabolism	map00360	22	29	161	7.15E-07
Starch and sucrose metabolism	map00500	18	60	277	2.58E-07
Valine, leucine and isoleucine biosynthesis	map00290	10	3	37	3.89E-03
Drug metabolism - cytochrome P450	map00982	9	10	66	7.02E-03
Metabolism of xenobiotics by cytochrome P450	map00980	8	11	72	1.83E-02
Nicotinate and nicotinamide metabolism	map00760	8	4	40	2.10E-02
Chemical carcinogenesis	map05204	8	10	74	4.56E-02
Pentose and glucuronate interconversion	map00040	6	27	122	1.52E-03
Limonene and pinene degradation	map00903	6	5	33	1.19E-02
alpha-Linolenic acid metabolism	map00592	5	10	45	3.57E-03

^a^Number of up- and down-regulated genes enriched in this pathway

^b^Number of unigenes annotated in this pathway

### Selection of candidate metabolic resistance genes

Based on the known mechanisms of metabolic resistance in weeds [1, 3, 8, 10], we speculated that DEGs related to metabolism and signaling pathways were more likely to be associated with metabolic resistance to TM. In the present study, we selected the overlapping genes that were upregulated in MRT_ST vs. MRC_SC (|log2(fold change) | > 1) had related functional annotations, including those encoding CYPs, GSTs, GTs, and ABC transporters (Table 5). In addition, contigs with predicted annotations related to oxidase, POD, esterase, and hydrolase in the above-mentioned comparison or an assigned GO function related to pesticide metabolism were also selected (Van Eerd et al., 2003). In total of 34 contigs were selected (Table 5) for validating by qRT-PCR. Among these contigs, eight were annotated to the CYP family, one to the GST family, two to the GT family, two to the uridine diphosphate glucuronosyltransferase family, eight to the ABC transporter family, four to the POD family, and 9 to other families. The low number of candidate GST contigs is supported by the lack of difference in GST activity between MR and S plants (unpublished data).

**Table 5:** Identification of the up-regulated unigenes annotated and related to metabolic resistance in *Myosoton aquaticum* by RNA-Seq and qRT-PCR (2^-ΔCt^)

Gene ID	PFAM ID	Function annotation	RNA-Seq		FoldChage: qRT-PCR (2^-ΔCt^)	
			Padj^a^	FoldChange (MRT_ST)	*P*-value^b^	RNA-Seq samples (MRT_ST)^c^
c47752_g3	PF00067.17	CytP450, CYP716B1	2.40E-02	2.41	1.81E-01	1.24(0.17)
c49980_g3	PF00067.17	CytP450, CYP71A21	2.37E-02	3.24	6.00E-03	0.56(0.14)
c31888_g1	PF00067.17	CytP450, CYP72A219	7.73E-05	2.16	1.00E-03	0.45(0.08)
c28525_g1	PF00067.17	CytP450, CYP734A1	6.32E-07	2.28	5.00E-03	1.94(0.2) **
c49866_g6	.	CytP450, CYP76C1	3.73E-02	2.19	3.67E-04	3.22(0.34)***
c44104_g1	PF00067.17	CytP450, CYP82A4	2.69E-02	3.4	7.10E-02	0.91(0.06)
c48448_g1	PF00067.17	CytP450, CYP86B1	4.16E-06	2.04	1.00E-03	1.27(0.03)**
c14606_g1	PF00067.17	CytP450, CYP94A1	2.67E-01	1.49	1.31E-01	1.46(0.25)
c50084_g2	PF00032.12	Cytochrome b6-f	1.68E-02	5.22	4.30E-02	0.85(0.08)
c49404_g2	PF01578.15	Cytochrome c	2.56E-02	5.17	9.04E-01	1.02(0.17)
c48737_g9	.	Cytochrome c1–2	1.72E-02	2.47	4.30E-02	0.54(0.12)
c27216_g1	PF13417.1	GST, T1	8.89E-01	1.09	4.40E-01	0.89(0.18)
c33248_g1	PF03552.9	Glycosyltransferase, GT28	1.94E-04	2.79	1.00E-03	0.13(0.01)
c33752_g1	PF00295.12	Glycosyltransferase, GT28	3.39E-02	3.26	3.00E-03	0.27(0.06)
c47986_g3	PF00201.13	UDP-glycosyltransferase, UGT73B3	4.04E-07	3.48	3.16E-01	0.89(0.13)
c32196_g1	PF00201.13	UDP-glycosyltransferase, UGT78D2	8.88E-01	1.07	1.30E-01	1.28(0.25)
c40202_g1	PF04577.9	Glucosamine transferase	6.23E-03	2.37	6.24E-01	0.95(0.16)
c30791_g1	.	ABC transporter, ABCB2	1.34E-04	2.15	2.00E-03	0.65(0.05)
c49741_g6	.	ABC transporter, ABCB2	6.75E-13	2.62	1.00E-03	2.29(0.2) **
c49337_g1	PF03468.9	ABC transporter, ABCB29	6.96E-04	2.8	9.20E-02	0.7(0.06)
c45895_g2	.	ABC transporter, ABCC10	1.05E-03	2.45	2.00E-03	1.96(0.2)**
c45895_g3	.	ABC transporter, ABCC10	5.12E-07	4.89	9.20E-02	0.76(0.04)
c50054_g1	PF00005.22	ABC transporter, ABCC10	9.18E-06	2.61	2.10E-02	2.16(0.53) *
c39205_g1	PF00664.18	ABC transporter, ABCC3	4.55E-11	2.16	7.50E-02	1.54(0.33) **
c47115_g3	PF00664.18	ABC transporter, ABCC8	2.97E-02	2.67	6.30E-02	0.67(0.04)
c37150_g1	PF00403.21	Peroxidase	5.01E-06	2.51	7.00E-03	0.59(0.13)
c44363_g1	PF00141.18	Peroxidase 5	7.23E-25	6.74	1.56E-04	0.38(0.07)
c33094_g1	PF00141.18	Peroxidase 57	3.49E-14	3.94	1.30E-02	1.93(0.34) *
c42442_g1	PF00141.18	Peroxidase 57	1.15E-06	1.79	2.39E-04	1.73(0.07) ***
c44336_g7	.	Oxidase	2.75E-02	4.2	1.00E-03	0.54(0.02)
c40277_g1	PF00657.17	Esterase	6.59E-23	3.88	2.00E-03	0.44(0.05)
c48393_g3	.	Esterase	2.56E-04	3.7	3.81E-01	0.85(0.22)
c35829_g1	PF00722.16	Hydrolase	1.24E-04	4.59	8.90E-02	0.82(0.11)
c50023_g1	PF10551.4	Hydrolase	2.51E-08	4.73	6.00E-02	1.12(0.05)

MR_T, resistant *M. aquaticum* L seedlings sprayed tribenuron-methyl; S_T, susceptible *M. aquaticum* L seedlings sprayed tribenuron-methyl

^a^The resulting p-value was adjusted and expressed as the padj by the Benjamini-Hochberg procedure for controlling the false discovery rate

^b^Means were separated using Fisher’s protected least significant difference (LSD) test at the 5% level of probability (from SPSS analysis)

^c^*P*-value of < 0.05, 0.01, 0.001 is indicated by *, **, and ***, respectively (from SPSS analysis)

### qRT-PCR validation of candidate metabolic resistance gene expression

The expression of 34 candidate contigs was validated by qRT-PCR using original RNA samples from MR_T and S_T. We found that 10 of the contigs had higher expression levels in MR as compared to S samples (Table 5), including four for CYP, four for the ABC transporter, and two for POD.

**Fig. 6:**
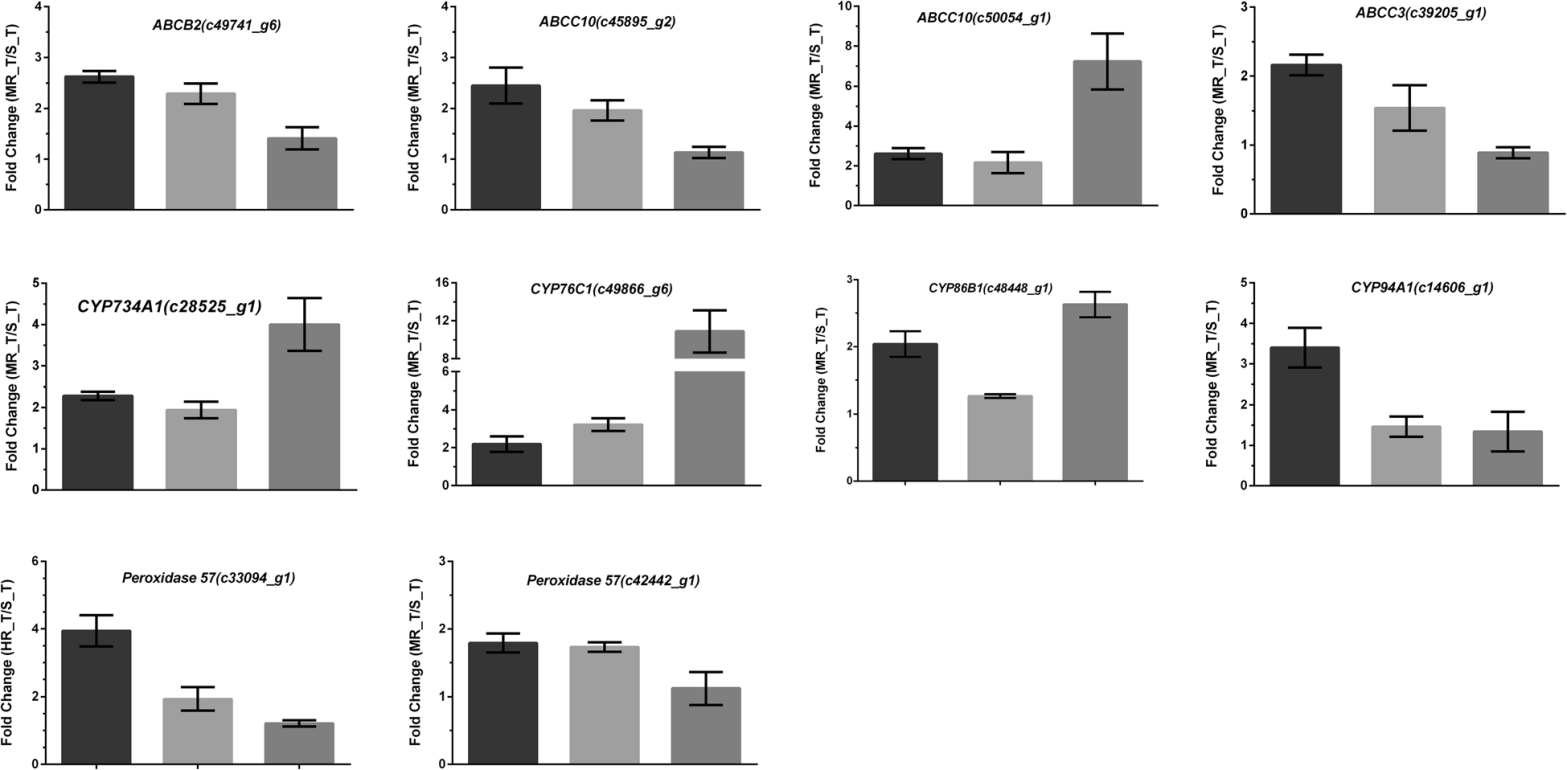
The qRT-PCR validations for the ten genes that showed up-regulated expression in *Myosoton aquaticum* samples. Dark bars for the RNA-Seq; grey bars for the qRT-PCR validation using the RNA-Seq samples; and dark grey bars for the qRT-PCR validation using the additional plant materials. Actin was used as the internal control gene. Means and SEs from three biological replicates are shown

We further examined the expression levels of the 10 candidate contigs in additional plants and found that four of these were more highly expressed in MR than in S plants (Table 5 and Fig. 6). Although there was some variation among individual plants, our analysis of the average expression levels of candidate contigs based on two rounds of qRT-PCR and RNA-Seq profiling revealed that three CYP and one ABC transporter were consistently and significantly overexpressed in MR (Fig. 6), including c28525_g1, c49866_g6, and c48448_g1 with homology to *CYP734A1*, *CYP76C1*, and *CYP86B1*, respectively, and c50054_g1 annotated as *ABCC10*.

## Discussion

In the present study, we identified an *M. aquaticum* population (MR) that had evolved moderate (6.15-fold) resistance to TM. We conclude that TM resistance in this population is due to CYP-mediated enhancement of herbicide metabolism rather than TSR based on the following lines of evidence: (1) ALS enzymes from S and MR plants exhibited similar in vitro sensitivity to TM; (2) *ALS* gene sequencing revealed no known ALS resistance mutations in resistant plants; and (3) treatment with the CYP inhibitor malathion reversed TM resistance. Malathion is known to inhibit the metabolism of sulfonylurea herbicides and thereby reverse metabolic resistance [7, 8]. Recent studies have indicated that metabolic resistance to ALS inhibitors is increasing among both monocot and dicot weed species, with co-occurrence of TSR frequently observed [1, 10, 32–35]. There have been few studies on metabolism-based herbicide resistance without TSR in broadleaf weed species. *M. aquaticum* is a broadleaf weed that is widespread in China, with many populations exhibiting resistance to the ALS inhibitors including TM. Identifying genes involved in NTSR can provide insight into the evolution of metabolic resistance in dicot weeds as well as a basis for the development of strategies to overcome this resistance.

The present study provides a transcriptomic resource for future studies. Given that NTSR mechanisms differ among and within weed species [3], we performed two RNA-Seq analyses: once for de novo assembly of the *M. aquaticum* transcriptome, and another to identify candidate contigs associated with TM in a specific MR population. The 101,307 assembled unigenes had an average size of 872 bp; this number of unigenes is higher and their average size is similar than those reported for other weed species [14, 18, 23–26, 36–41].

Genes that were differentially expressed between untreated resistant and susceptible plants were more revealing than those detected after herbicide treatment. However, some studies have shown that upregulation of gene expression induced by ALS inhibitors is an important resistance mechanism [3, 21, 23]. A commercial formulation of TM was used in the present study because any allele(s) enabling individual plant survival to herbicide must be strongly selected by repeated application of both the herbicide and associated formulations under realistic field conditions [3]. Taking into consideration differences in genetic background and herbicide application in the two populations from different locations, we selected constitutive as well as herbicide-induced genes related to herbicide metabolism that were differentially expressed between MR and S biotypes as candidate genes involved in TM resistance in *M. aquaticum*.

In total, 10 contigs were selected as candidate NTSR genes (Table 5) presumed to encode proteins with homology to four CYPs (CYP734A1, CYP76C1, CYP86B1, and CYP94A1), four ABC transporters (ABCB2, ABCC10, ABCC3, and ABCC10), and two PODs (POD57). The qRT-PCR analysis confirmed that four of the contigs (c28525_g1 [CYP734A1], c49866_g6 [CYP76C1], c48448_g1 [CYP86B1], and c50054_g1 [ABCC10]) were expressed at consistently higher levels in MR than in S plants (Table 5 and Fig. 6). Variable expression of validated NTSR-related genes has also been reported in *L. rigidum* [19, 20], *Echinochloa phyllopogon* [42], *D. sophia* [26], *A. aequalis* [23], and many other weed species. NTSR in individual plants may arise from slightly different sets of resistance genes [26]. In our study, *CYP734A1*, *CYP76C1*, *CYP86B1*, and *ABCC10* were identified as the major candidate genes conferring metabolic resistance to *M. aquaticum*.

CYPs play a critical role in the metabolism of secondary metabolites and catalyze diverse reactions in plants [43]. CYP-mediated herbicide metabolism has been well-characterized in resistant *L. rigidum* and *E. phyllopogon*, and several CYP isoforms were shown to confer metabolic resistance to ALS inhibitors and other herbicides via different modes of action [8, 10]. CYP81A12 and CYP81A21 in *E. phyllopogon* metabolize penoxsulam and bensulfuron-methyl [42]; CYP72A31 in *A. thaliana* metabolizes bispyribac sodium and bensulfuron-methyl [44]; and CYP94A1 in common vetch (*Vicia sativa* L.) contributes to plant defense against chemical injury [45]. Transgenic *A. thaliana* expressing CYP76B1 and CYP76C subfamily members show enhanced resistance to monoterpenols and phenylurea herbicides [46]. The CYP734A family in plants typically functions in the catabolism of brassinosteroids, which protect against herbicide toxicity [47, 48]. The CYP86A and CYP86B subfamilies catalyze hydroxylation of fatty acids (Compagnon et al. 2009; Pinot and Beisson 2011); CYP-mediated hydroxylation and epoxidation are also important pathways for herbicide degradation in plants (Siminszky 2006; Werck-Reichhart et al. 2000). In addition, CYP71AK2, CYP71A4, CYP72A254, CYP734A6, CYP86B1, CYP94A1, CYP94A2, CYP96A13, and CYP96A15 are highly expressed in ALS inhibitor-resistant *E. phyllopogon* [49], *A. aequalis* [23], *D. sophia* [26], and *Echinochloa colona* [50]. Moreover, CYPs associated with herbicide tolerance have been identified in crops such as CYP71C6v1 in wheat [51]; CYP81A6 and CYP72A31 in rice [44, 52]; CYP71A10 in soybean [53, 54]; CYP76B1in Jerusalem artichoke [55]; and CYP71A11 and CYP81B2 in tobacco [56]. Notably, a previous study reported that purified recombinant CYP71C6v1 expressed in wheat metabolized sulfonylurea herbicides, including bensulfuron-methyl, metsulfuron-methyl, chlorsulfuron, triasulfuron, and TM [51]. Thus, the CYP genes identified here in the MR population may play an important role in TM metabolism and resistance in *M. aquaticum*.

GST and GT are two other families of herbicide-detoxifying enzymes that contribute to NTSR [57–59]. GST and GT are associated with NTSR to ACCase and ALS inhibitors in the grass weeds *L. rigidum* and *A. myosuroides* [18–21]. In addition, the overexpression of two GST genes (*GST-T3* and *-F1*) and two GT genes (*GT83A1* and *GT75D1*) has been linked to the mesosulfuron-methyl resistance in the grass weed *A. aequalis* [23]. In the present study, one GST and five GT contigs were annotated; however, the expression of the corresponding genes did not differ significantly between MR and S plants (Table 5), suggesting that they do not contribute to TM resistance in *M. aquaticum*. The lack of involvement of GST and GT in ALS inhibitor resistance in dicot weeds has also been documented in TM-resistant *D. sophia*, and may be attributable to (1) distinct ALS herbicide resistance genes in grass and broadleaf species; (2) differences in the metabolism of specific ALS herbicides; (3) variable recombination resulting from self- and cross-pollination; and (4) insufficient data.

Plant ABC transporter families are among the largest and most diverse, and have been implicated in the detoxification of xenobiotics, including herbicides [60, 61]. However, in contrast to CYP, GST, and GT gene families that are involved in metabolic resistance to herbicides, ABC transporters function by compartmentalizing herbicides and/or their metabolites [43]. In the present study, one contig (c50054_g1) was annotated to *ABCC10* and may be associated with TM resistance. Similar results were recently reported in several weed species: *ABCC1* in a resistant *D. sophia* population was implicated in TM resistance [26]; *ABCC8* and *ABCB11* were linked to metabolic resistance to mesosulfuron-methyl in multi-herbicide-resistant *A. aequalis* [23]; *ABCB10* was upregulated in a resistant *B. syzigachne* population exhibiting NTSR to fenoxaprop-P-ethyl [22, 23]; and several ABC transporters were highly expressed in glyphosate-resistant horseweed (*Conyza canadensis*) [62, 63] and *Lolium perenne* [25], as well as in paraquat-resistant goosegrass (*Eleusine indica* L.) [14]. In fact, ABC transporter activity towards herbicide metabolites is well established in plants and protects against toxicant injury. The ABC transporter AtABCC1/AtMRP1 exhibits enzymatic activity towards the GS-conjugated herbicide metolachlor, whereas AtABCC1 and AtABCC2 in *A. thaliana* mediate tolerance to arsenic and arsenic-based herbicides [64]. The ABC transporter AtOPT6 in *A. thaliana* is associated with resistance to the herbicide primisulfuron [65]. In addition, overexpression of *AtPgp1*—a multi-drug resistance gene family member—and its garden pea homolog *psNTP9* have been shown to confer resistance to multiple herbicides in *A. thaliana* [66]. It is thus likely that *ABCC10* contributes to metabolic resistance to TM in *M. aquaticum*.

Peroxidase, oxidase, esterase, and hydrolase families are implicated in NTSR [1]. In the present study, we identified nine upregulated contigs potentially encoding proteins with homology to these enzymes; however, their expression levels did not differ significantly between MR and S plants in validation experiments (Table 5). Similar to these results, a previous study reported that POD is unlikely to be involved in TM resistance in the broadleaf weed *D. sophia* [26]. We propose that POD, oxidase, esterase, and hydrolase genes are unlikely to be associated with TM resistance in *M. aquaticum*.

## Conclusion

An *M. aquaticum* population (MR) moderately resistant to TM was identified in this study, and was found to exhibit NTSR rather than TSR, which is likely due to CYP-mediated metabolic resistance and ABC transporter-mediated sequestration of metabolites. The contigs c28525_g1 (with homology to *CYP734A1*), c49866_g6 (with homology to *CYP76C1*), c48448_g1 (with homology to *CYP86B1*), and c50054_g1 (with homology to *ABCC10*) are potential NTSR genes or markers for metabolic resistance to TM in *M. aquaticum*. Other genes potentially involved in herbicide resistance may be discovered by analyzing other resistant *M. aquaticum* populations. The functional characterization of these genes using yeast and *A. thaliana* transgenic systems is now underway; future studies should investigate the regulation of metabolic resistance genes to elucidate the NTSR network in weeds. Our findings provide insight into the molecular basis of metabolic herbicide resistance mechanisms in broadleaf weed and a basis for developing weed management strategies to overcome this major threat to crop production.

## Methods

### Plant materials

Mature seeds of resistant *M. aquaticum* HN10 were harvested from winter wheat fields in Zhumadian, Henan Province (32°58′16″N, E 114°38′54″), in 2013. Seeds collected from at least 30 mature plants randomly distributed in the field were thoroughly mixed, air dried, and stored in paper bags at 4 °C until use. A previously documented herbicide-susceptible population (HN03, designated S) originating from Xixian, Henan Province (32°19′08.16″N, 114°45′09.62″E), served as the control in the present study [28].

Prior to planting, seeds were germinated on 0.6% (*w*/*v*) plant agar in an artificial chamber (20 °C/15 °C, 12:12-h light/dark cycle), and seedlings were transplanted into plastic pots (20 per pot) filled with potting mix (50% organic matter and moist loam soil, pH 5.6). The pots were randomly distributed in a greenhouse under controlled conditions (natural sunlight, 25 °C/15 °C, ~ 75% relative humidity), with no fertilizer supplementation and watering every 48 h. The seedlings were thinned to 10 uniform plants per pot at the 2–3 leaf stage, were treated with herbicides when they reached the 3–4 leaf stage. The herbicides were sprayed using a compressed-air moving-nozzle cabinet sprayer equipped with a TeeJet 9503EVS flat fan nozzle and calibrated to deliver 450 L ha^− 1^ at 275 kPa. After the treatment, the plants were returned to the greenhouse and the number of survivors (plants with no new growth were considered as dead) or biomass (dry weight of above-ground material) was recorded 3 weeks after herbicide treatment.

Twice-selected resistant plants derived from HN10 were used for experiments. Briefly, a single-rate screen test was performed to identify resistant plants as described in our previous study [27]. Plants that survived treatment with 22.5 g ai ha^− 1^ (2-fold higher than the field recommend rate) TM (75% WG; DuPont, Juxing, Shanghai, China) were selected for *ALS* gene sequencing. Resistant plants with no known ALS resistance mutations were isolated before flowering and F1 seeds were obtained by cross pollination. The seeds were germinated and cultivated for a second round of TM resistance selection, and *ALS* sequencing was performed as described above to obtain F2 seeds (designated MR).

### Effect of malathion on TM resistance

Whole-plant response experiments were carried out to determine the sensitivity of S and MR populations to TM in the absence and presence of malathion as described in our previous study [29]. The malathion application rate was 750 g ai ha^− 1^, and there was no negative effect on *M. aquaticum* seedling growth. TM was applied at 0, 0.016, 0.08, 0.4, 2.0, 10, and 50 g ai ha^− 1^ to S and MR populations. The above-ground material was harvested 21 days after herbicide treatment and the dry weight was recorded. There were three replicates per treatment, and the experiment was performed twice.

### In vitro ALS inhibition assay

Seedlings from the S and MR populations were harvested at the 3–4 leaf stage. ALS extraction and the in vitro herbicide inhibition assay were performed as previously described [67, 68]. ALS activity was determined colorimetrically (530 nm) with a ultraviolet spectrophotometer (Thermo Fisher Scientific, Waltham, MA, USA) by measuring acetoin production. Three subsamples from each extraction were assayed, with two extractions per population.

### Statistical analysis

Values from repeated experiments were subjected to one-way analysis of variance (*P* ≤ 0.05); since there were no statistically significant differences between repeats, the data were pooled and subjected to non-linear regression analysis using SigmaPlot v.12.5 (Systat, San Jose, CA, USA). The herbicide rate or dose resulting in 50% growth reduction (GR_50_) or 50% inhibition of ALS activity (I_50_) was estimated using a four-parameter log-logistic model:

$$ Y=C+\frac{D-C}{1+{\left(x/{ED}_{50}\right)}^b} $$

where *C* is the lower limit, *D* is the upper limit, *b* is the slope at ED_50_, and ED_50_ is the effective dose causing a 50% reduction. The resistance index was calculated by dividing the GR_50_ or I_50_ value of the resistant population by that of the sensitive population.

### *ALS* gene sequencing

Genomic DNA was extracted from shoot tissue of individual plants at the 3- to 4-leaf stage using the Plant Genomic DNA kit (Tiangen Biotech, Beijing, China) according to the manufacturer’s instructions. The primers used to amplify the *M. aquaticum ALS* gene fragment containing five conserved regions (A to E) and PCR protocol have been described elsewhere [69, 70]. PCR products were purified with 1.0% agarose gels using the TIANgel Midi Purification kit (Tiangen Biotech) and were sequenced from both ends by Shanghai Sangon Biological Engineering and Technology Service Co. (Shanghai, China). Sequence data were aligned and compared using DNAMAN v.8.0.8 software (Lynnon Biosoft, San Ramon, CA, USA).

### Sample collection and preparation for RNA-Seq

The confirmed resistant (MR) and susceptible (S) *M. aquaticum* populations were cultivated and treated with herbicide as described above and 24 h later, leaf materials were collected from treated MR and S as well as untreated control plants. The experimental design included three biological replicates of MR and S for the untreated control and TM (T; 11.25 g ai ha^− 1^) treatments, for a total of twelve 10-seedling combinations of the MR and S populations. Collected samples (three biological replicates × two treatments × two populations) were immediately frozen in liquid nitrogen and stored at − 80 °C until RNA extraction.

### RNA extraction, library construction, sequencing, and bioinformatics analysis

Total RNA extraction and quality control were performed according to standard protocols [23]. cDNA library construction and Illumina sequencing were performed by Annoroad Gene Technology Co. (Beijing, China); 150-bp paired-end reads were obtained using an HiSeq 4000 platform (Illumina, San Diego, CA, USA). Raw reads (transformed by the Consensus Assessment of Sequence and Variation (CASAVA, v.1.8.2)) containing the adapter poly-N (> 5% of the unknown sequences designated as “N”) and low-quality reads (> 15% bases with quality value < 20) were filtered out using in-house Perl scripts to obtain high-quality clean reads. De novo transcriptome assembly was conducted in Trinity v.20140717 [71] under its default parameter values, to obtain the transcripts and unigenes-the longest transcript of a set of transcripts that appears to originate from the same transcription locus. To evaluate the accuracy of assembly, all unigenes were matched to the sequences of *Arabidopsis thaliana* (L.) Heynh, using the BLAST-Like Alignment Tool (BLAST) with E ≤ 1e-20. The bioinformatics analysis strategy for obtained unigenes is detailed in our previous study [23]. Briefly, after de novo transcriptome assembly, the possible open reading frames for each unigene were identified with TransDecoder v.20140717 [72]. To obtain comprehensive, functional information regarding the sequences, unigenes were annotated using seven publicly available databases (Table 3) by means of local BLAST programs (NCBI, NIH, Bethesda, USA) which used a significance threshold of E < 1e-5. In addition, Trinotate v.20140717 software was used to identify protein domains, predict signal peptides, and locate transmembrane regions [73–75]. Gene Ontology (GO, http://www.geneontology.org/) annotation of unigenes was performed with Blast2GO [76], and GO functional classification was carried out using WeGO software (http://wego.genomics.org.cn) [77]. Pathway assignment was performed using the Kyoto Encyclopedia of Genes and Genomes (KEGG) Automatic Annotation Server (http://www.genome.jp/kegg/kaas/).

### Analysis of differentially expressed genes (DEGs)

cDNA libraries were constructed for three biological replicates each of MR and S samples with TM treatment (referred to as MR_T and S_T, respectively) and six corresponding control samples (MR_C and S_C, respectively). Quality assessment and sequencing were carried out for the 12 samples as described above. Gene expression levels were calculated based on reads per kilobase million mapped reads (RPKM) [78]. Differences in the abundance of each read between sample pairs (MR_C vs. S_C, MR_T vs. S_T, MR_T vs. MR_C, S_T vs. S_C, and MR_T vs. S_C) were calculated using DESeq 2 v.1.4.5 [79]. The resultant *P* values were converted to q values using the Benjamini-Hochberg procedure to minimize the false discovery rate [80]. Genes with q < 0.05 and |log2(fold change) | ≥ 1 were identified as DEGs, and were further analyzed by GO and KEGG enrichment analysis with the hypergeometric test [81], in which the P value was adjusted for multiple comparisons to a q value and genes in the whole genome served as background data. GO or KEGG terms with q < 0.05 were considered as significantly enriched.

### Identification of candidate resistance genes and verification of relative expression levels

To verify the reliability of expression data, 34 candidate resistance genes were selected and their levels were quantified by qRT-PCR using the primers listed in Additional file 6.

Total RNA was extracted and purified using the RNApre Pure Plant kit (Tiangen Biotech), and 1 μg was used for first-stand cDNA synthesis with the FastQuant RT Super Mix Kit (Tiangen). qRT-PCR was performed in 96-well plates on a LightCycler 96 system (Roche, Basel, Switzerland) using SuperReal PreMix Plus (SYBR Green) (Tiangen). The 20 μL reaction mixture contained 10 μL of 2× SuperReal PreMix Plus, 1 μL diluted cDNA, 0.6 μL primers, and 7.8 μL RNase-free ddH_2_O, with three replicates prepared of each cDNA sample. The reaction conditions were as follows: 95 °C for 15 min, followed by 40 cycles of 95 °C for 10 s, 60 °C for 20 s, and 72 °C for 20 s. At the end of the reaction, melting curve analysis was performed to confirm that there was no non-specific amplification; similar amplification efficiencies were observed for the target and internal control genes (90.0–103.7%). Fold change in gene expression was calculated with the comparative cycle threshold (CT) method (2^−ΔCt^) [82] relative to samples from susceptible plants, where ΔCT = [CT target gene − CT actin]. Data were normalized against to the expression level of actin (GenBank accession no. KP099106.1), a housekeeping gene. Three biological replicates of each treatment were prepared. The qRT-PCR data were analyzed with the Student’s *t*-test (*P* < 0.05) using SPSS software (SPSS Inc., Chicago, IL, USA).

The expression patterns of unigenes whose relative expression levels were verified by qRT-PCR were in accordance with RNA-Seq profiles were also confirmed in additional samples (seed germination, plant cultivation, herbicide treatment under the same conditions, and RNA extraction following RNA-Seq) collected at 72 h after TM treatment.

## Additional files

Additional file 1: Quality assessment of the reads generated from RNA-Seq for the resistant (MR) and susceptible (S) *Mysoton aquaticum* populations. (XLSX 10 kb)

Additional file 2: Length distribution of unigenes characterized from the RNA-seq libraries of *Myosoton aquaticum*. (PDF 49 kb)

Additional file 3: Species distributions of the BLASTX matches of the *Myosoton aquaticum* transcriptome unigenes. (PDF 6 kb)

Additional file 4: The annotation for unigenes of *Myosoton aquaticum. (XLSX 14194 kb)*

Additional file 5: The respective numbers of overlapping up-regulated genes in comparative treatment groups. (XLSX 10 kb)

Additional file 6: Primer pairs used for the qRT-PCR relative quantification of gene expression in *Myosoton aquaticum*. (DOCX 19 kb)

## Abbreviations

ABC: ATP-binding cassette
ACCase: Acetyl-coenzyme A carboxylase
BLAST: Basic Local Alignment Search Tool
BP: Biological process
CASAVA: Consensus Assessment of Sequence and Variation
CC: Cellular component
CYP: Cytochrome P450 monooxygenase
DEGs: Differentially expressed genes
GO: Gene Ontology
GST: Glutathione-S-transferase
GT: Glycosyltransferase
KEGG: Kyoto Encyclopedia of Genes and Genomes
MF: Molecular function
NCBI: National Center for Biotecnology Information
NTSR: Non-target site resistance
POD: Peroxidase
qRT: quantitative real-time
RNA-Seq: RNA-sequencing
RPKM: Reads per kilobase million mapped reads
SRA: Sequence read archive
TM: Tribenuron-methyl
TSR: Target-site resistance
