# Supplementary figures and images for: Non-target site-based resistance to tribenuron-methyl and essential involved genes in Myosoton aquaticum (L.)
Source: BMC Plant Biol. 2018 Oct 11;18:225. doi: 10.1186/s12870-018-1451-x (PMC6180388; doi:10.1186/s12870-018-1451-x)

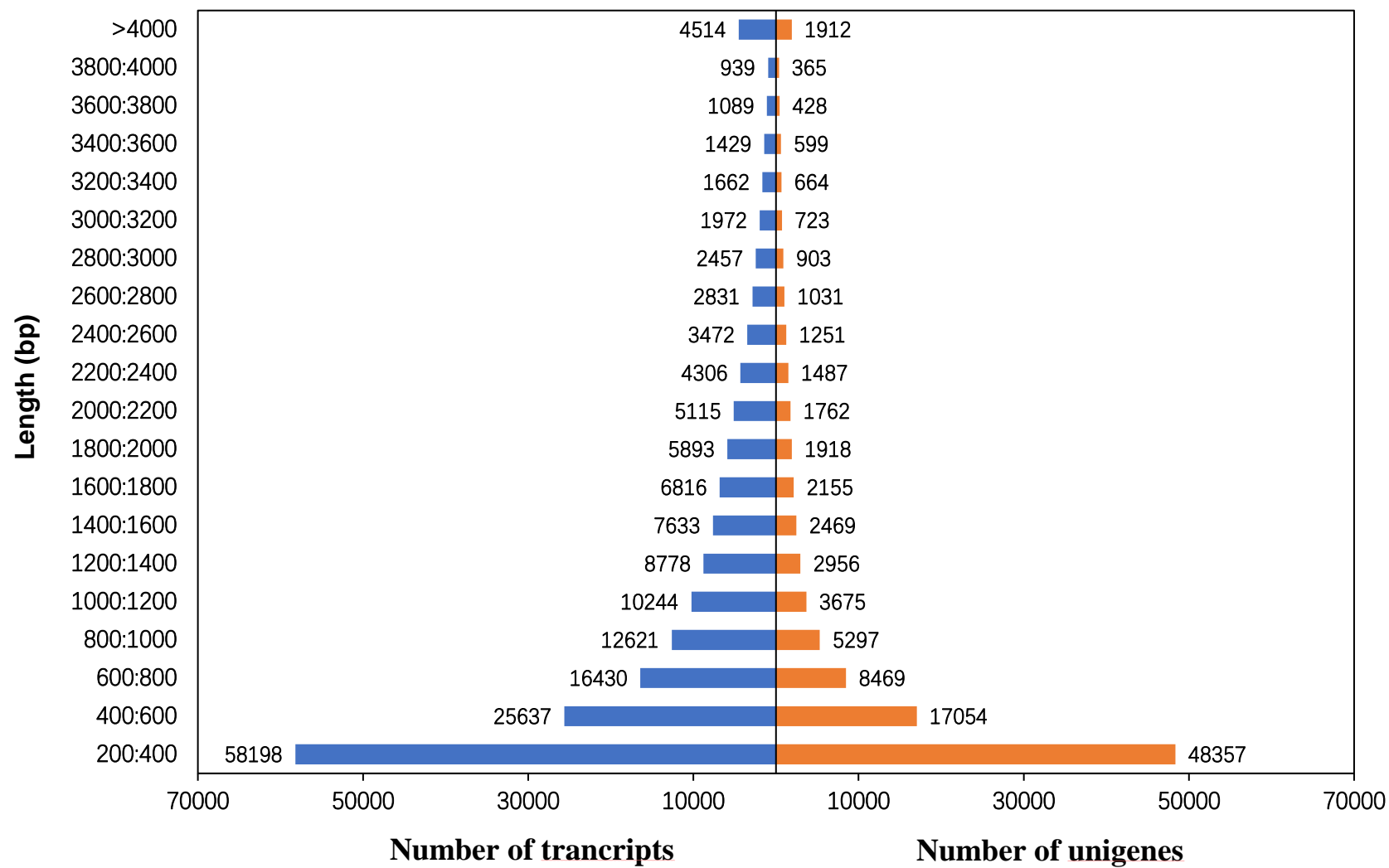

Supplement: Supplementary file 2 — Length distribution of unigenes characterized from the RNA-seq libraries of Myosoton aquaticum. (PDF 49 kb) [file 12870_2018_1451_MOESM2_ESM.pdf]

Species Distribution

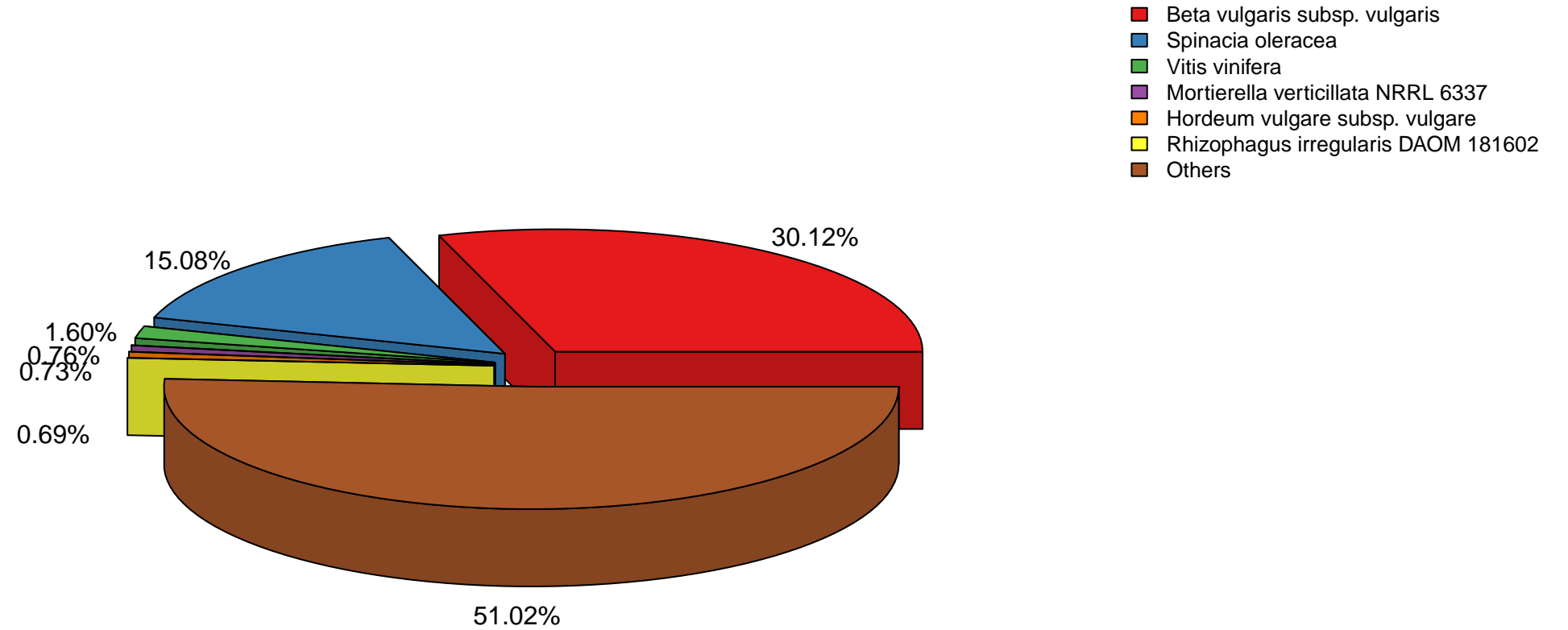

Supplement: Supplementary file 3 — Species distributions of the BLASTX matches of the Myosoton aquaticum transcriptome unigenes. (PDF 6 kb) [file 12870_2018_1451_MOESM3_ESM.pdf]
