# Supplementary material for: Non-target site-based resistance to tribenuron-methyl and essential involved genes in Myosoton aquaticum (L.)
Source: BMC Plant Biol. 2018 Oct 11;18:225. doi: 10.1186/s12870-018-1451-x (PMC6180388; doi:10.1186/s12870-018-1451-x)
Supplement: Supplementary file 6 — Primer pairs used for the qRT-PCR relative quantification of gene expression in Myosoton aquaticum. (DOCX 19 kb) [file 12870_2018_1451_MOESM6_ESM.docx]

Supplementary Table S3. Primer pairs used for the qRT-PCR relative quantification of gene expression in *Myosoton aquaticum*.

| Gene ID | Gnen annotation | Primers | Sequence (5' to 3') |
| --- | --- | --- | --- |
| c47752_g3 | CYP716B1 | F | TTCTCATTTGGATTGACCCTTG |
|  |  | R | GAAGGGAATGGACCAACACC |
| c49980_g3 | CYP71A21 | F | GCCTAAGTAGACCAACATAAGCG |
|  |  | R | ACCACCATCTCCACCAAAACTAC |
| c31888_g1 | CYP72A219 | F | GAATGACCCGTTCTCCAAGTC |
|  |  | R | CCCTGTTTTGCGTACTACATCC |
| c28525_g1 | CYP734A1 | F | GAGGATTTGAGGACGATTTCTG |
|  |  | R | GAACCTGATGGAGGGCACC |
| c49866_g6 | CYP76C1 | F | AAACAAGGCACAGCCAAGC |
|  |  | R | TTTTCACAAGTTCAGCCACAGAG |
| c44104_g1 | CYP82A4 | F | TCATCGTCTTCTTGTACCCTCC |
|  |  | R | CAGGATTTCAAAAGCATTACGG |
| c48448_g1 | CYP86B1 | F | TCTCAGGAAGGGCAATACGG |
|  |  | R | TCCAACGGTTTTCCTTAGACG |
| c14606_g1 | CYP94A1 | F | GCCGTTACGATAATCTTCCG |
|  |  | R | GCCCACCAACATACCCTTTC |
| c50084_g2 | Cytochrome b6-f | F | ATAGACGTTGAGGCGGACG |
|  |  | R | ATCCGACCCTTATGGACTAACC |
| c49404_g2 | Cytochrome c | F | AGGACCAAGAGGTATCCACCG |
|  |  | R | TCACTCCGATTTCTTCCATTTC |
| c48737_g9 | Cytochrome c1-2 | F | TCCGTTCTCCAGTTTCTTTGC |
|  |  | R | TCGGGGAAGTAAGAGTTGTAGTG |
| c27216_g1 | GSTT1 | F | AATCCTCAAGAAGCTGTTGAATG |
|  |  | R | TTTCTCCTCCTCGTCTAATACCTC |
| c33248_g1 | GT28 | F | CGGCATTACTCCCAGGTTG |
|  |  | R | GGAAGGTGCTTGGAACGATG |
| c33752_g1 | GT28 | F | TTTTAGCACTTTGTTTTGTCTTGG |
|  |  | R | CGTCTACTCTACTTCCTCCACTCC |
| c47986_g3 | UGT73B3 | F | AGGAAAAGAAGAAGATGAAGGAGA |
|  |  | R | AATGAGTCACAAACCCACTAACC |
| c32196_g1 | UGT78D2 | F | ATTTGGTGAAGCATTTTAGCG |
|  |  | R | GCTGAACGAAGGAAAGACGAG |
| c40202_g1 | Glucosamine transferase | F | CCACAGCCTTGCATCTTCG |
|  |  | R | TCGCACTGTATCATTCCTTCG |
| c30791_g1 | ABCB2 | F | TCTTCTTGACTTCAGCCCTCG |
|  |  | R | GTGGAAACGCCTTTTCTCG |
| c49741_g6 | ABCB2 | F | TCAGGACGGGATGGGTAGC |
|  |  | R | GCTCCAGATGTATCGGCATTC |
| c49337_g1 | ABCB29 | F | GTGCCCAGCAGGAACGAC |
|  |  | R | CTGGACCACGGTTCACAGG |
| c45895_g2 | ABCC10 | F | TTTGTTTAGGGCGTGCTTTG |
|  |  | R | CACCAATCTCCCGTCATCG |
| c45895_g3 | ABCC10 | F | CGAGTTTTGTCCGTTCTTGC |
|  |  | R | AGTCGGGTCCTCATTTTCTTC |
| c50054_g1 | ABCC10 | F | CCGTAACCAATGAACACCTCC |
|  |  | R | GAAAAAGTAGCAATCTGCGGTG |
| c39205_g1 | ABCC3 | F | AGAAGACTCTTAGGGACGAAACG |
|  |  | R | AGTTATACTCCGCCACCTTGC |
| c47115_g3 | ABCC8 | F | TTTATCAGTTGGTCCAGTCCTCC |
|  |  | R | TGTTTACTATCCTTGCGACCTTG |
| c37150_g1 | Peroxidase | F | TCCACAACTTGGTGACCGTAG |
|  |  | R | TGGACATTGGTACTAGAATTGAGG |
| c44363_g1 | Peroxidase 5 | F | CGTAATCACTTCAATGGCACC |
|  |  | R | ACCTCCGATGAAACCCTTATG |
| c33094_g1 | Peroxidase 57 | F | CTGTAAACGATGGACCAGGAAG |
|  |  | R | GTGCCGATGTTGTTACGCTC |
| c42442_g1 | Peroxidase 57 | F | TGAGTATGCCAATACGAGAAGC |
|  |  | R | AACGGTATGCCCTCCTGTG |
| c44336_g7 | Oxidase | F | CAAATCCCGTGAACATCATACC |
|  |  | R | GCGTTTAGTTTGTTGACTTGAGAG |
| c40277_g1 | Esterase | F | ATGAATTGCTACGCTAACCTCG |
|  |  | R | TGGGCTTCCACTTTTGTCC |
| c48393_g3 | Esterase | F | CCAACCACCCCTGAACTCC |
|  |  | R | TCATGGGTGAAATCGGAGG |
| c35829_g1 | Hydrolase | F | CACAAGATTTCCACAACTACGC |
|  |  | R | TCGGCTTTGTATTTCCCATC |
| c50023_g1 | Hydrolase | F | GGGGAAGTTTGCCGAGTTG |
|  |  | R | CGCCTTTGGCAAGTTTTCTC |
| β-actin | Reference gene | F | CCATTGAACCCTAAGGCTAACAG |
|  |  | R | CCACTAGCATACAAGGAAAGAACG |
